# Examining Medical Students’ and Professionals’ Perspectives on Eye Donation and Corneal Transplantation

**DOI:** 10.7759/cureus.67757

**Published:** 2024-08-25

**Authors:** Rekha R Mudhol, Nakshatra H Bullapur

**Affiliations:** 1 Ophthalmology, Shri Basanagouda Mallanagouda (BM) Patil Medical College, Bijapur Lingayat District Educational University (BLDE) (Deemed to be University), Vijayapura, IND

**Keywords:** medical students' attitudes, organ donation awareness, medical community awareness, eye donation, corneal transplantation

## Abstract

Background

Corneal blindness affects millions across India. Corneal transplantation is the primary treatment; however, there is a severe shortage of donor corneas. This study aimed to assess awareness, knowledge, and attitudes towards corneal donation among medical students and professionals, identify barriers to donation, and explore popular information sources for promoting awareness about corneal transplants and eye donation.

Methods

Using a validated, self-administered questionnaire, this cross-sectional study surveyed 350 medical undergraduates, postgraduates, and doctors. Data collection occurred over a period of three months via Google Forms (Mountain View, CA: Google LLC). The questionnaire assessed knowledge about corneal donation criteria, willingness to donate corneas, factors influencing donation decisions, and sources of awareness about corneal transplants. Ethical clearance was obtained, and data was analyzed using SPSS version 20.0 (Armonk, NY: IBM Corp.).

Results

Regarding donor eligibility, 269 (76.9%) knew that anyone regardless of age can donate, 270 (77.1%) knew that donation occurs postmortem, 269 (68.3%) knew the time limit for cornea collection, only 133 (38%) knew medical conditions impacting donor eligibility, and 250 (71.4%) knew only corneal tissue is retrieved. A large number, 333 (95.1%), are aware of the functions of eye banks. Of the participants, 228 (65.1%) were willing to donate their corneas. Popular reasons for donations included helping blind people improve their quality of life and the nobility of the act. The main barriers include a lack of awareness, a desire to preserve the body, health issues, and ethical concerns during extraction. The most preferred sources of information are social media, health workers/eye camps, TV advertisements, and celebrity endorsements. Overall, doctors and postgraduates demonstrated superior knowledge in most areas compared to undergraduates, and ophthalmologists showed higher knowledge in specific domains (like age limits, factors affecting the eligibility of eye donors, time frame for cornea harvesting, and the identification of certain diseases and conditions barring donation) than non-ophthalmologists.

Conclusion

Our research uncovered knowledge deficits regarding corneal donation criteria and procedures mainly among undergraduate students. The findings highlight the importance of impactful educational initiatives that utilize preferred communication channels. These efforts should address myths and misconceptions, analyze vital motivating factors, overcome barriers, and understand cultural perspectives influencing the overall attitude towards corneal donation.

## Introduction

India has the world’s largest corneal-blind population [1]. Around 6.8 million people in India suffer from less than 6/60 vision in one eye due to corneal-related diseases [2]. Common causes of corneal blindness include infections, such as bacterial, viral, fungal, and protozoal, as well as conditions like keratoconus, dry-eye disease, and trachoma. Important predisposing factors are trauma, excessive use of contact lenses, and steroid medication usage [3].

Corneal transplantation is the primary sight-restoring procedure for correcting corneal blindness [4]. However, due to the anatomical and physiological factors of the eye, corneal tissue must be harvested from donors. Different types of corneal transplants and keratoplasty exist, such as penetrating keratoplasty, deep anterior lamellar keratoplasty, and endothelial keratoplasty.

The National Programme for Control of Blindness (NPCB) data indicates that there are 1,20,000 people in India affected by corneal blindness and awaiting corneal transplants. Of the 2,50,000 corneas needed per year, only 25,000 are donated. This leads to a grave shortage of more than 2,25,000 corneas required for transplantation [5]. Many barriers contribute to this deficiency, such as lack of transparency regarding cornea usage, lack of trust, risk of upsetting family members, need for the body to remain intact after death, fear of mistreatment of the body, and religious factors [6]. As a result, there is a need for increasing awareness among the public and medical professionals.

The primary objective of this study was to assess the level of awareness and knowledge regarding corneal transplants among medical students and professionals, evaluate the attitude and willingness to donate cornea among the study group and collect information about common reasons for not donating eyes. The goal was also to assess the attitude and willingness to donate cornea among the given study group and to collect information about common reasons for not donating eyes. We also aimed to gain data about different sources of information through which awareness of corneal transplants and eye donation is spread.

## Materials and methods

This cross-sectional observational study was conducted over three months in 2023. The sample comprises 500 medical undergraduates, postgraduates, and medical professionals specializing in the ophthalmology and non-ophthalmology branches. The questionnaire was distributed to three to five colleges including our institution. The colleges and participants were selected through purposive sampling, collaborative outreach, and direct dissemination to individuals through personal networks. The questionnaire was distributed to approximately 500 eligible participants, out of which 350 responded. The data was collected anonymously using a pre-tested (done via conducting a thorough literature review and consulting with ophthalmologists from the university to form and select the questionnaire items), self-administered questionnaire using Google Forms (Mountain View, CA: Google LLC) in English. Participants were asked to undertake the survey on their personal electronic devices through the Google Forms weblink provided to them after obtaining their consent to participate in the study. Consent was obtained through two methods. For participants who were approached directly by the researchers, explicit or verbal consent was obtained prior to providing the survey link (verbal consent was deemed appropriate due to minimal risk to participants and to maintain anonymity). For participants from other healthcare institutions and medical colleges, implied consent was assumed through their voluntary participation in the online survey after accessing the shared link. Those who declined were excluded from the study. No prior information about the subject was provided through physical, electronic, or audio-visual means.

The questionnaire collects demographic details of the participants such as ages, designation, and department. The participants were given 10 questions to assess their knowledge, awareness, and attitude towards eye donation and corneal transplant. The questions were derived from previously existing published literature on corneal donation (the table in the appendix) [6-9]. The 10 multiple-choice questions (a combination of open and close-ended questions) covered various areas regarding donors, such as age limit, time of cornea harvesting, eligibility, parts of the eye used, and functions of eye banks. Additionally, it gauged the willingness of the participant to donate their eyes, the factors influencing their decision, and the best sources of spreading awareness. The correct responses were decided based on established medical facts and current international and national guidelines on corneal donation. The questionnaire was thoroughly reviewed by the faculty of the Department of Ophthalmology at Shri Basanagouda Mallanagouda (BM) Patil Medical College to confirm the validity of the questions used in the study. The reliability of the questionnaire was assessed through data sources and methodological triangulation to enhance overall reliability. The level of knowledge and awareness was assessed based on the participants’ selection of the correct response from the provided options. Scores were calculated according to the accuracy of these selections. The questionnaire follows a combination of categorical and ordinal scaling methods. Ethical approval for the study was obtained from the Ethical Committee of Bijapur Lingayat District Educational University (BLDE) (Deemed to be University). Approval number is #BLDE(DU)/IEC/1006/2023-24.

Inclusion criteria

We primarily included undergraduate medical students currently enrolled in a medical college, postgraduate medical students or residents in any specialty, and practicing doctors with a valid medical license. Participants from both public and private medical institutions were included and all the participants were aged 18 years and above. We ensured that only those willing to participate in the study were included and informed consent was obtained.

Exclusion criteria

Our study excluded non-medical healthcare professionals (e.g., nurses, pharmacists), medical professionals who had previously participated in a similar study on corneal donation awareness, and individuals who were unable to comprehend or complete the survey instrument. Participants with incomplete survey responses were excluded (for data quality purposes) and those who withdrew consent during the study were also not included.

Sample size

With anticipated mean±SD of knowledge score corneal transplant 5.24±1.9 s, the study would require a sample size of a minimum of 350 subjects with a 95% level of confidence and a precision of 0.2 [6]. The formula n = z^2^ × S^2^/d^2^ was used to calculate the required sample size for our study. Here, Z is the statistic at α level of significance, d^2^ is absolute error, S is the common standard deviation, and q is 100-p.

Statistical analysis

The data obtained were entered into a Microsoft Excel sheet (Redmond, WA: Microsoft Corp.), and statistical analysis was performed using SPSS version 20 (Armonk, NY: IBM Corp.). Results of continuous variables were presented as mean±SD and categorical variables as frequency and percentages. The association between categorical variables was compared using the chi-square test. P-value <0.05 was considered statistically significant. All statistical tests were performed using a two-tailed approach.

## Results

Out of the 350 participants who responded (response rate 70%), 174 were undergraduates and interns, 93 were postgraduates from various departments, and the remaining 83 participants were consultants, doctors, or medical professionals. Most participants were between 21 and 30 years of age. Forty-one participants were under the age of 20 years, 30 were aged 31-40 years, 24 were aged 41-50 years, 22 were aged 51-60 years, and six of the candidates were 61 years and older. Among professionals and postgraduates/residents, 42 were from ophthalmology, and 134 were from non-ophthalmology. The demographic details (age, designation, and department) of the participants have been mentioned in Table 1.

**Table 1:** Demographic details of participants (age, designation, and department).

Variables		Number of participants (%)
Age of the participants (in years)	≤20	41 (11.7)
	21-30	227 (64.9)
	31-40	30 (8.6)
	41-50	24 (6.9)
	51-60	22 (6.3)
	61+	6 (1.7)
Designation	Professional	83 (23.7)
	Post-graduate	93 (26.6)
	Undergraduate	174 (49.7)
Department	Ophthalmology	134 (38.3)
	Non-ophthalmology	42 (12.0)
	Undergraduate	174 (49.7)

Knowledge and awareness about corneal donation

Regarding the age limit for corneal donation, 91.6% of professionals, 80.6% of residents, and 67.8% of students answered these questions correctly that anyone can donate and that age is not a limiting factor. The correct response is that anyone can donate. Forty-two (12%) answered that elderly individuals aged above 65 years are not eligible for donation while the remaining 39 (11.1%) participants believe that children and adolescents aged under 18 years are not allowed for corneal donation (Table 2).

**Table 2:** What age group cannot donate cornea? *The correct response. P-value <0.05 is considered statistically significant. The p-values assess the association between the respondents' designation and their awareness of corneal donation eligibility (chi-square test=19.008; p=0.001).

Designation	Anyone can donate (%)*	<18 years (%)	>65 years (%)
Professionals	76 (91.6)	4 (4.8)	3 (3.6)
Residents/postgraduates	75 (80.6)	8 (8.6)	10 (10.8)
Medical students	118 (67.8)	27 (15.5)	29 (16.7)
Total (n=350)	269 (76.9)	39 (11.1)	42 (12)

The majority of Indian medical professionals correctly understood that only deceased individuals could donate corneas. Specifically, 86.7% of professionals, 80.6% of residents, and 70.7% of students responded accurately that corneas could only be donated after death. While 80 participants (22.9%) incorrectly assumed that donations are also accepted from living donors (Table 3).

**Table 3:** When can you donate your eyes? *The correct response. P-value <0.05 is considered statistically significant. The p-values assess the association between the respondents' designation and their understanding of when eye donation can occur (chi-square test=9.098; p=0.011).

Designation	Only after death (%)*	Living people can also donate (%)
Professionals	72 (86.7)	11 (13.3)
Residents/postgraduates	75 (80.6)	18 (19.4)
Medical students	123 (70.7)	51 (29.3)
Total (n=350)	270 (77.1)	80 (22.9)

Regarding the ideal 4-6 h window for cornea harvesting after death, in our study, 67 (80.7%) professionals and 73 (78.5%) residents provided the correct answer, while only 99 (56.9%) students answered accurately. The correct answer is 4-6 h, which is the preferred time frame for corneal extraction (Table 4).

**Table 4:** When can a cornea be harvested? *The correct response. P-value <0.05 is considered statistically significant. The p-values examine the association between the respondents' designation and their knowledge of what time frame corneas can be harvested (chi-square test=21.872; p=0.001).

Designation	4-6 hours (%)*	24-48 hours (%)	Anytime after death (%)
Professionals	67 (80.7)	6 (7.2)	3 (3.6)
Residents/postgraduates	73 (78.5)	9 (9.7)	2 (2.2)
Medical students	99 (56.9)	36 (20.7)	7 (4)
Total (n=350)	239 (68.3)	51 (14.6)	12 (3.4)

According to our study, the majority of professionals (68.7%) stated that individuals with certain conditions, such as AIDS, hepatitis, rabies, and others, could not donate corneas. In comparison, only 37 residents (39.8%) and 39 students (22.4%) held the same belief. Another important finding was that a huge number of participants incorrectly believed that conditions like wearing glasses, astigmatism, diabetes, and hypertension also hamper eligibility for donation. The correct answer in this case is that people who have AIDS, hepatitis, certain cancers, and rabies, among other diseases, are ineligible for donation (Table 5).

**Table 5:** Who is not eligible to donate their eyes? *The correct response. P-value <0.05 is considered statistically significant. The p-values evaluate the association between the respondents' designation and their awareness of who is not eligible for eye donation and the medical conditions that affect it (chi-square test=72.601; p=0.0).

Designation	(a) Everyone can donate (%)	(b) People suffering from AIDS, hepatitis, certain cancers, rabies, etc (%)*	(c) People who wear spectacles, have astigmatism, hypertension, diabetes, history of cataract surgery, or died in road traffic accidents (%)	(d) Both b and c (%)
Professionals	12 (14.5)	57 (68.7)	2 (2.4)	12 (14.4)
Residents/postgraduates	24 (25.8)	37 (39.8)	0 (0.0)	32 (34.4)
Medical students	22 (12.6)	39 (22.4)	6 (3.4)	107 (61.4)
Total (n=350)	58 (16.6)	113 (38)	8 (2.3)	151 (43.1)

According to our research, when it comes to knowledge about parts of the eye used for donation, 81.9% of doctors, 74.2% of residents, and 64.9% of students answered that only the cornea is extracted. In comparison, 21.4% of all participants stated that the cornea and other parts are extracted per the requirements, and some (7.1%) believed that the entire eye is taken. While the sclera and other parts of the eye are also harvested worldwide, our study considered only corneal donation as the correct response, which is the predominant practice in India. While scleral tissue is used globally, not all Indian facilities have the infrastructure for harvesting and processing. To maintain simplicity and relevance to the Indian context, we focused exclusively on corneal donation. Thus, here only the cornea being harvested is considered the correct response (Table 6).

**Table 6:** What part of the eye is used for donation? *The correct response. P-value <0.05 is considered statistically significant. The p-values analyze the association between the respondents' designation and their knowledge of which part of the eye is harvested for donation (chi-square test=14.320; p=0.006).

Designation	Only cornea (%)*	Cornea and other required parts like lens, conjunctiva, etc. (%)	Entire eye (%)
Professionals	68 (81.9)	9 (10.8)	6 (7.2)
Residents/postgraduates	69 (74.2)	15 (16.1)	9 (9.7)
Medical students	113 (64.9)	51 (29.3)	10 (5.7)
Total (n=350)	250 (71.4)	75 (21.4)	25 (7.1)

When it came to understanding the function of eye banks, 333 (95.1%) participants answered that eye banks are used for checking the eligibility of donors and collecting, storing, and evaluating corneal tissues. The correct answer is all of the above (Table 7).

**Table 7:** Do you know the functions of eye banks? *The correct response. P-value <0.05 is considered statistically significant.

Function(s)	No. of participants (%)
Checking eligible donors	3 (0.9)
Collection and storage of donated eyes	13 (3.7)
Evaluating the tissue donated	1 (0.3)
All of the above*	333 (95.1)
Total	350 (100)

Attitude toward eye donation

The study assessed the attitudes and willingness of participants to donate their own eyes. Among the subjects, 65.1% responded positively, indicating their willingness to donate eyes. However, 3.4% directly declined, while 31.4% remained undecided. Among this, 71% of residents and 75.9% of professionals among the 65.1% who expressed a willingness to donate said yes. Students, however, demonstrated relatively lower willingness, with only 56.9% indicating they would donate their eyes (Table 8).

**Table 8:** Are you willing to donate your eyes? The p-values assess the association between the respondents' designation and their willingness to donate their own eyes (chi-square test=16.091; p=0.003).

Designation	Yes (%)	No (%)	Not decided (%)
Professionals	63 (75.9)	1 (1.2)	19 (22.9)
Residents/postgraduates	66 (71)	0 (0.0)	27 (29)
Medical students	99 (56.9)	11 (6.3)	64 (36.8)
Total (n=350)	228 (65.1)	12 (3.4)	110 (31.4)

The current study found that among those willing to donate their eyes, the primary motivations were providing better vision for others (48%) and eye donation being a noble act or service to humanity (33.1%). Other reasons include a desire to contribute towards medical research (5.1%) and feeling inspired by others (2.3%). However, 11.4% of those willing to donate declined to provide a reason (Table 9).

**Table 9:** Reasons to donate eyes.

Reason	Number of participants (%)
Help blind people get better vision and improve quality of life	168 (48)
Inspired by a family member/friend/movie/documentary	8 (2.3)
It is a noble act/service for humanity	116 (33.1)
Want to help in medical research	18 (5.1)
Declined	40 (11.4)
Total	350 (100)

When asked about possible reasons why participants may be apprehensive about organ donation, 86 participants (24%) provided answers. Among these 24%, the top reasons were lack of awareness (5.4%), wanting the body to remain intact after death (3.4%), health problems (2.9%), no trust in the healthcare system (2.3%), not knowing how the eyes will be used (2.6%), family objections (2.6%), and religious reasons (2%). A majority of the participants, i.e., 264 (75.4%) declined to provide any answers (Table 10).

**Table 10:** Reasons to not donate your eyes.

Reasons	Number of participants (%)
Afraid that my body parts will be sold illegally	2 (0.6)
Decreased eyesight	1 (0.3)
I don't know how they will use my cornea	9 (2.6)
I don't trust the Indian healthcare system	8 (2.3)
I have health problems	10 (2.9)
I want my body to remain intact after death	12 (3.4)
Lack of awareness about eye donation	19 (5.4)
Mistreatment of my body/may disfigure my face	4 (1.1)
My family will say no	9 (2.6)
Personal reasons	4 (1.1)
Religious reasons	7 (2)
Not answered	264 (75.4)
Total	350

Table 11 shows that participants believe social media, health workers/eye camps, and TV advertisements are the most popular channels for spreading awareness. Interestingly, people under 30 and those above 40-60 preferred social media, while most of the 31-40 age group believed TV advertisements to be the better mode for creating awareness.

**Table 11:** Preferred source of information among different age groups. *The preferred options/highest responses of the group. P-value <0.05 is considered statistically significant. The p-values assess the association between the respondents' designation and their opinions on the most effective mode of spreading awareness about eye donation (chi-square test=51.187; p=0.009).

Age (in years)	Celebrity endorsements (%)	Health workers/eye camps (%)	Newspapers (%)	Posters (%)	Public campaigns (%)	Social media (%)	T. V. advertisements (%)	Total No of participants
≤20	2 (4.9)	8 (19.5)	1 (2.4)	2 (4.9)	3 (7.3)	16 (39)*	9 (22)	41
21-30	17 (7.5)	45 (19.8)	3 (1.3)	1 (0.4)	15 (6.6)	116 (51.1)*	30 (13.2)	227
31-40	6 (20)	4 (13.3)	2 (6.7)	0 (0.0)	1 (3.3)	8 (26.7)	9 (30)*	30
41-50	2 (8.3)	1 (4.2)	0 (0.0)	0 (0.0)	5 (20.8)	10 (41.7)*	6 (25)	24
51-60	3 (13.6)	3 (13.6)	0 (0.0)	1 (4.5)	2 (9.1)	9 (40.9)*	4 (18.2)	22
61+	0 (0.0)	2 (33.3)	0 (0.0)	0 (0.0)	2 (33.3)	0 (0.0)	2 (33.3)	6

Comparison of the knowledge and performance levels of ophthalmologists vs. non-ophthalmologists in eye donation awareness study

The study categorized medical professionals and residents based on their specialization fields, distinguishing between ophthalmology and non-ophthalmology professionals. Out of 350 participants, 134 were non-ophthalmology residents and professionals, and 42 were ophthalmology residents and professionals. Regarding the age limits for cornea donation, 97.7% of ophthalmologists provided the correct response, higher than the 82.1% of non-ophthalmology physicians (chi-square=20.968, p=0.000). Similarly, 93% of ophthalmologists answered accurately that only deceased individuals could donate corneas, exceeding the 80.6% rate among non-ophthalmology physicians (chi-square=11.360, p=0.003). For the optimal 4-6 h time frame after death to harvest corneas, 86% of ophthalmologists answered correctly vs. 77.6% of non-ophthalmologists/physicians (chi-square=24.549, p=0.000). Ophthalmologists (86%) also outperformed general physicians (42.5%) in identifying specific diseases and conditions that can render someone ineligible as an eye donor (chi-square=81.119, p=0.0001). While 83.7% of ophthalmologists stated that only the cornea is extracted during donation, some noted that newer techniques allow for procuring other required eye parts. This contrasted with 76.1% of non-ophthalmology participants indicating only the cornea was taken (chi-square=16.537, p=0.002 is considered statistically significant). The data reveals that ophthalmologists and ophthalmology residents demonstrated higher accurate knowledge levels than non-ophthalmology medical professionals.

## Discussion

Regarding the age limit for corneal donation, our study demonstrates better awareness among our participants while a study conducted by Gopal et al. in England found that a considerably smaller proportion of residents and junior doctors (28%) knew there was no corneal donation age limit [7]. This stark difference highlights a potential gap in education or awareness between residents and postgraduates in our study’s location and those in England. Interestingly, in research from Brazil, a majority of professionals (77%) held the misconception that age was a significant limiting factor for corneal donation eligibility [8]. This finding contrasts our results with those from the English study, suggesting that misconceptions about age restrictions in corneal donation may vary widely across countries and healthcare systems.

Our study revealed that most Indian medical students (70.7%) understood that corneas could only be donated posthumously. In contrast, research conducted by Okoye et al. in Nigeria found that many students (72.5%) were unaware that corneas can only be harvested from deceased donors [9]. Similarly, a study from Malaysia revealed an even more extensive knowledge gap, with only a tiny fraction of students (33%) aware that corneal donation is only possible after death [10]. This stark difference suggests that Indian students in our study better understood this crucial aspect of corneal donation compared to their Nigerian and Malaysian counterparts.

Regarding the time frame for corneal harvesting, we observed similar trends when comparing our findings to studies from other regions. In our study, professionals (80.7%) exhibited a better level of knowledge compared to professionals tested in Delta State, Nigeria (39.8%) with less than half being aware of the optimal time for eye donation [11]. Similarly, a study by Gopal et al. in England found that only a small proportion of residents and junior doctors (35.7%) were aware of the time limit for cornea harvesting which is significantly lower compared to our residents (78.5%) [7]. However, a study conducted in Bagalkot presented a different picture [12]. There, medical students demonstrated a remarkably high level of awareness regarding the time frame for cornea harvesting after death (93%), surpassing the knowledge levels observed in our study (56.9%) and other international studies. These comparisons highlight a consistent pattern across different regions as follows: medical students often better understand the time limit for corneal viability compared to practicing professionals and residents. It also suggests that while this information may be effectively taught in medical schools, there might be a need for continued education and refresher courses for practicing professionals and residents.

Our study also revealed a significant disparity in knowledge regarding corneal donation eligibility among medical professionals and students. Professionals (68.7%) demonstrated a substantially higher level of understanding compared to residents (39.8%) and students (29.3%) when it came to identifying conditions that preclude corneal donation, such as certain cancers, AIDS, hepatitis, and rabies. Interestingly, a significant portion of all participants (43.1%) in our study held misconceptions about corneal donation eligibility. Many incorrectly believed that conditions like wearing glasses, astigmatism, diabetes, and hypertension would disqualify potential donors. Comparatively, in Brazil, just over half of the doctors (53%) correctly identified infectious diseases as a contraindication for donation [8]. This indicates a slightly lower level of awareness compared to the professionals and residents in our study. The study conducted in England revealed that while only a small proportion of residents (37.8%) were aware that blood cancers make donors ineligible, which is in line with our results, and a more significant portion of the English residents (62.9%) correctly identified blood-borne infections as a disqualifying factor [7]. Contrastingly, a Nigerian study focusing on medical students showed a high level of awareness (84%) regarding AIDS as a contraindication for corneal donation [9]. This finding shows that there is significantly lower awareness among students in our study. These comparisons highlight the variation in knowledge about corneal donation eligibility across different countries. This is particularly important due to the consequences of misinformation in this field, which can impact corneal donation rates.

Our research revealed similar knowledge among medical professionals and students regarding the parts of the eye extracted during corneal donation. Doctors demonstrated the highest level of understanding (81.9%), followed by residents (74.2%). Students showed a slightly lower level of awareness (64.9%). These findings show that a majority can identify that only the cornea is retrieved during donation. When comparing our findings, we observed a significant difference in knowledge levels among Malaysian students, where only 26.21% were aware that corneas are exclusively used during eye donation procedures [10]. This comparison highlights a relatively higher level of understanding among Indian medical students than their Malaysian counterparts. It also highlights the importance of an internationally standardized medical curriculum to ensure consistent and accurate information is provided to medical students.

When it came to understanding the function of eye banks, an overwhelming majority (95.1%) of participants correctly answered that eye banks are used for checking the eligibility of donors and collecting, storing, and evaluating corneal tissues. In contrast to a study by Nekar conducted in Hubli, Karnataka, where 74.1% of respondents were aware that eye banks collect eyes, these findings show a higher level of awareness about the functions of eye banks [13]. This shows adequate understanding and knowledge about eye banks among the medical fraternity, although better education can be provided regarding the locations and names of local eye banks.

In our study, we also assessed the attitudes and willingness of participants to donate their own eyes. A significant number of subjects (65.1%) responded positively, indicating their willingness to donate eyes. Residents and professionals demonstrated a higher willingness to donate corneas than students. While these findings reveal a reasonably positive attitude towards eye donation, some hesitancy is still noted among the participants, especially the students. The lower rates among students suggest a need for increased awareness and motivational efforts targeting this group.

When asked about the reasons for donating eyes, the primary motivations were providing better vision for others (48%) and eye donation being a noble act or service to humanity (33.1%). Other reasons included a desire to contribute to medical research and feeling inspired by others. These findings align with the results from Singh et al.’s research in Delhi on medical students, where the main reasons stated were the nobility involved in the act (85%) and the pleasure of helping people who are blind (78.3%) [14]. Across these studies, the desire to help others and the sense of nobility of eye donation emerged as decisive motivating factors.

We also asked about possible reasons why participants may be apprehensive about organ donation. Out of the 86 participants (24%) who answered, the top reasons were lack of awareness, wanting the body to remain intact after death, health problems, family objections, and not knowing how their eyes will be used. Others also included no trust in the healthcare system and religious reasons. This is similar to the findings from Singh et al.’s Delhi study, where 32.7% of medical student respondents cited a lack of awareness as the main reason for not donating eyes [14]. Similarly, Hussen et al.’s research in North West Ethiopia found the following reasons: the participants required more information (40.8%), wanting to be buried with an intact body (28%), religious reasons (15%), family refusal (5.2%), and concerns about body mistreatment (4.8%) [15]. Similar results were found in various other studies [16,17]. Another study conducted in South India revealed that 63% of respondents stated family objection as a reason for refusal [18]. Across these studies, lack of awareness and informational gaps emerged as prominent barriers to eye donation. Cultural factors like desires for an intact body after death, religious beliefs, and family objections also played a significant role in the unwillingness.

When asked about their preferred sources for receiving information on eye donation, out of the 350 participants, the most popular choice was social media (45%). Health workers/eye camps, TV advertisements, celebrity endorsements, public campaigns, newspapers, and posters followed this. Further analysis by age groups revealed that social media remained the top preference across most age brackets, except for those aged 31-40 years who preferred receiving information through TV ads. These findings highlight the dominance of social media as the preferred information source for eye donation awareness among older and younger generations. However, traditional channels like health workers, TV advertising, and celebrity influences still emerged as noteworthy methods for spreading awareness. Understanding these preferred information sources can help guide more targeted awareness campaigns according to specific audiences.

Compared to non-ophthalmologists, ophthalmologists demonstrated better levels of knowledge and awareness regarding age limits, factors affecting eligibility of donors, time frames for corneal harvesting, and the identification of certain diseases and conditions barring donation. While most ophthalmologists (83.7%) stated that only the cornea is extracted during donation, some also noted that newer techniques allow for procuring other required eye parts. This difference in knowledge levels is likely attributable to their specialized training and expertise in the field. However, the data also reveals potential gaps in some areas among non-ophthalmology medical professionals. This shows the critical need for widespread education initiatives to enhance knowledge about eye donation within the medical community.

Strengths and limitations****

Our study reflects participants’ genuine, unassisted knowledge, thus accurately representing their current understanding without external influences. Additionally, by not providing visual aids or information videos before attempting the questions, there was minimal risk of introducing bias or influencing the responses. The study offers a comprehensive and diverse sample of the medical community, including professionals/doctors, residents, and medical students, and showcases different levels of medical education and experience. Some limitations of the study include that participants may only reliably recall some of the information they have acquired regarding eye donation, which could lead to an underestimation of their actual knowledge. Due to self-reporting bias, participants might overestimate or underestimate their knowledge or attitudes due to social factors. Without providing background information, the study may not accurately capture how participants would respond if given proper information or education through reading materials or visual aids before answering questions. The results may not accurately indicate levels of knowledge among medical professionals or students in different regions or healthcare systems.

## Conclusions

Our study concluded that there are gaps in knowledge among the medical community regarding eligibility age, living vs. deceased donation, optimal time for corneal harvest, and medical conditions that preclude donation. Overall, the knowledge level of professionals and residents is better than that of students. The study revealed that the overall willingness to donate eyes was 65.1%, with the highest rates observed among professionals and the lowest among students. These results underscore the importance of targeted awareness campaigns, particularly focusing on educating and motivating students to increase eye donation rates. The top motivations were providing vision for others and the nobility of the act. Key deterrents included lack of awareness, cultural beliefs about keeping the body intact, distrust in medical systems, and family objections. Social media emerged as the preferred information source across most age groups, except the 31-40 years age group, who preferred TV advertisements. Traditional methods, like health workers and celebrity endorsements, also had significance. The findings highlight opportunities to improve education around eye donation policies and counter misinformation. It is essential to utilize preferred information channels strategically in campaigns aimed at specific age groups and addressing cultural concerns to increase eye donation rates.

In brief, our study revealed gaps in knowledge about eye donation eligibility and processes among the medical community, particularly students. It also highlighted the need for targeted awareness campaigns using preferred information channels to address motivations, deterrents, and cultural beliefs impacting overall willingness to donate eyes.

## Appendices

**Table 12:** Questionnaire used in this study.

Variables	Questions	Options									
Demographic details	Age (years)	-	-	-	-	-	-	-	-	-	-
	Designation	a. Undergraduate	b. Postgraduate	c. Consultant	-	-	-	-	-	-	-
	Department	a. Ophthalmology	b. Non- ophthalmology	-	-	-	-	-	-	-	-
Questions assessing knowledge and awareness	1. What age group cannot donate cornea?	a. <18 years	b. >65 years	c. Anyone can donate	-	-	-	-	-	-	-
	2. When can you donate your eyes?	a. Only after death	b. Living people can also donate	-	-	-	-	-	-	-	-
	3. When can a cornea be harvested?	a. 4-6 hours	b. 24-48 hours	c. Within 72 hours	d. Any time after the death	-	-	-	-	-	-
	4. Who is not eligible to donate their eyes?	a. Everyone can donate	b. People suffering from AIDS, hepatitis, certain cancers, rabies, etc.	c. People who wear spectacles, have astigmatism, hypertension, diabetes, or had cataract surgery, died in road traffic accidents	d. Both b and c	-	-	-	-	-	-
	5. What part of the eye is used for donation?	a. Entire eye	b. Only cornea	c. Cornea and other required parts like lens, conjunctiva, etc.	-	-	-	-	-	-	-
	6. Do you know the functions of eye banks?	a. Collection and storage of donated eyes	b. Checking eligible donors	c. Evaluating the tissue donated	d. All of the above	-	-	-	-	-	-
Questions assessing attitude and willingness	7. Are you willing to donate your eyes?	a. Yes	b. No	c. Not decided	-	-	-	-	-	-	-
	8. If yes, why do you want to donate your eyes?	a. It’s a noble act/service for humanity	b. Help people get better vision and improve quality of life	c. Inspired by a family member/friend/movie	d. Want to help in medical research	e. Others	-	-	-	-	-
	9. If no, then why not?	a. Religious reasons	b. Lack of awareness (regarding eye donation)	c. I don’t trust the Indian healthcare system	d. I don’t know how they will use my cornea	e. Mistreatment of my body/may disfigure my face	f. My family will say no	g. I want my body to remain intact after death	h. Afraid my body parts will be sold illegally	i. I have health problems	j. Others
	10. Which source of information do you believe is the best mode of spreading awareness (e.g., TV, social media, public campaigns)?	a. TV advertisements	b. Social media	c. Celebrity endorsement	d. Newspapers	e. Public campaigns	f. Posters	g. Health workers/eye camps	-	-	-
